# Physicochemical, Structural, Thermal and Rheological Properties of Flour and Starch Isolated from Avocado Seeds of Landrace and Hass Cultivars

**DOI:** 10.3390/molecules27030910

**Published:** 2022-01-28

**Authors:** Ndahita De Dios-Avila, Juan Manuel Tirado-Gallegos, Claudio Rios-Velasco, Gregorio Luna-Esquivel, Néstor Isiordia-Aquino, Paul Baruk Zamudio-Flores, Mario Orlando Estrada-Virgen, Octavio Jhonathan Cambero-Campos

**Affiliations:** 1 Doctorado en Ciencias Biológico Agropecuarias, Universidad Autónoma de Nayarit, Km. 9, Carretera Tepic-Compostela, Xalisco C.P. 63780, Mexico; ndahitadedios@gmail.com; 2 Facultad de Zootecnia y Ecología, Universidad Autónoma de Chihuahua, Periférico Francisco R. Almada Km 1, Chihuahua C.P. 31453, Mexico; jtirado@uach.mx; 3 Fisiología y Tecnología de Alimentos de la Zona Templada, A.C.-Unidad Cuauhtémoc, Centro de Investigación en Alimentación y Desarrollo, Avenida Río Conchos s/n, Parque Industrial, Apartado Postal 781, Ciudad Cuauhtémoc, Chihuahua C.P. 31570, Mexico; pzamudio@ciad.mx; 4 Unidad Académica de Agricultura, Universidad Autónoma de Nayarit, Km. 9, Carretera Tepic-Compostela, Xalisco C.P. 63780, Mexico; gollole@hotmail.com (G.L.-E.); nisiordia@uan.edu.mx (N.I.-A.); estra0288@gmail.com (M.O.E.-V.)

**Keywords:** flour, starch, X-ray diffraction, *Persea americana*, functional properties

## Abstract

The objective of this study was to obtain and characterize flours and starches from the avocado seeds of Hass and landrace cultivars. The morphological, physical-chemical, structural, thermal and rheological characteristics were evaluated. The flour yield of the Hass and landrace cultivars was 41.56 to 46.86% (*w*/*w*), while for starch, it was 35.47 to 39.57% (*w*/*w*) (cv. Hass and landrace, respectively). Scanning electron microscopy (SEM) revealed the presence of oval starch granules and other particles in flour, in contrast to flours, starches showed lower ash, proteins and lipids content. However, the amylose content was higher in starches (42.25–48.2%). Flours showed a higher gelatinization temperature (*T_p_* = 73.17–73.62 °C), and their starches presented greater gelatinization enthalpy (∆*H_gel_* = 11.82–13.43 J/g). All samples showed a B-type diffraction pattern, and the crystallinity was higher in the flours. The rheological analysis (flow curves and viscoelastic tests) evidenced a pseudoplastic (*n* = 0.28–0.36) behavior in all samples analyzed, but the consistency index (*k*) was higher in starches. In general, the flours and starches from avocado seeds presented interesting proximal, thermal and functional properties for possible application in food systems, and these findings could contribute to the revaluation of this by-product.

## 1. Introduction

The avocado (*Persea americana* Miller) is one of the main perennial crops in Mexico and currently ranks first place in worldwide production, with around 2 million tons (t) per year [1]. More than 54% of its production is exported to the USA, Canada and Japan; the rest is destined for the local market for fresh consumption, guacamole and oil production [2]. The pulp is used for human consumption, while the husk and seed are discarded. In this regard, Dabas, et al. [3] mention that 2700 t is discarded annually; however, these agroindustrial wastes can be used or transformed to generate products or by-products with commercial value. The revaluation of wastes from different industries has become one of the main economic approaches for zero-wastes industrial activity [4,5,6]. Few studies can be found regarding the use of these residues, and they have mainly focused on obtaining phytochemical compounds [7], oils [8], natural dyes [3], bacterial culture media [9] and starch [10,11,12,13,14,15,16,17,18]. 

Mexico has a wide diversity of cultivars, such as Boot, Choquette, Bacon, Grana, Fuerte, Native (commonly known as landrace or autochthonous), Zutano and Hass [19]. Depending on the cultivar, avocado seeds can represent approximately 16% of the fresh fruit weight and are mainly constituted by water (~50%), starch (up to 29%), fiber (3%), protein (2.5%), sugar (2.5%) and ash (1.4%), among other components [12,13,20]. It is possible to obtain flours from avocado seeds; however, little information exists about the potential applications in the agri-food industry [20,21]. 

On the other hand, starch is a biopolymer consisting of amylose and amylopectin, isolated from various conventional sources, especially cereals and tubers [22]. This biopolymer presents a wide versatility in food and industrial applications; it is is used as a thickening, encapsulating, gelling and texturizing agent [23]. There is a great interest in exploring non-conventional botanical starch sources, which allow materials with similar properties or higher than those found in conventional sources [12]. In this sense, different cultivars, such as Zutano, Mantequilla, Daysi and Hass, have been explored as alternative starch sources. The Hass avocado is the most studied cultivar for starch isolation, due to the commercial importance of its fruits. In addition, the type of cultivar, agroclimatic conditions and the maturity of the fruits are factors that strongly influence the morphological, structural, thermal and rheological properties of isolated starches [17,18,24,25,26].

Therefore, further studies are required to determine the properties and possible applications of avocado-seed flours and starches. In this context, the aim of this study was to isolate and characterize the physicochemical, structural, thermal and rheological properties of the flours and starches from the avocado seeds of Hass and landrace cultivars. 

## 2. Results and Discussion

### 2.1. Yield, Proximate Analysis and Apparent Amylose Content

The yields in flour and starch (dw) from avocado seeds cv. Hass and landrace are shown in Table 1. Flour yields cv. Hass (46.86%) and landrace (41.56%) showed significant differences between both cultivars. There were compositional differences; for example, the moisture content of landrace avocado seeds is higher than cv. Hass (58.43 > 53.14%, wet base, data not shown) [27]. These results were similar to those reported by Rivera, et al. [18], with a yield of 46.2% in avocado-seed flours cv. Hass, but differ from those reported by Mahawan, et al. [21] and Dos Santos, et al. [28] in avocado-seed meal cv. Hass (32.9%) and cv. Daysi (36.7%), respectively. 

On the other hand, the starch yield was 39.57 and 35.47% for the cv. Hass and landrace, respectively. These values are within the range (20 to 42%) reported in the starch yields from avocado cultivars, such as Hass, Zutano, Mantequilla and Daisy [12,13,15,16,18]. The variation between these yields may be associated with the starch-isolation methods. Compared to starches obtained from other by-products, the starch yields from the avocado seeds of cv. Hass (39.57 wt%) and landrace (39.57 wt%) were similar to the value reported by Oliverira et al. 2018 in mango kernels Tommy Atkins (38.5 wt%) [29]. On the other hand, avocado-starch yields were higher than those registered for litchi seeds (12.16 wt%) [30] and jackfruit (16.9 wt%) [31]. The proximate analysis of flours and starches from avocado seeds cv. Hass and landrace is shown in Table 1.

The chemical composition between the flours of both cultivars showed no significant variation, especially in the content of ash (2.5%) and protein (5.2%). These results are similar to those recorded by Rivera, et al. [18] for flour from avocado seed meal cv. Hass (ash, 1.79%; lipids, 2.71%; protein, 6.70%). The lipid content was higher in the flour of cv. Hass (2.42%); this variability in its composition could be influenced by the avocado cultivar and its ripening stage [32]. In this regard, Ceballos and Montoya [27] mention that the lipid content tends to increase during the growth of the fruit and the ripening process. 

The protein content was lower than that reported by Mahawan, et al. [21] for avocado flour (7.75%). Based on these values, avocado-seed flours could be used to produce crunchy foods, such as snacks, since flours with a high protein content, such as wheat (12.4%), are more appropriate for elaborating bakery products with a spongy texture and appearance [33]. On the other hand, the avocado-seed flours can be used as a protein source for animal feed or agricultural supplementation [7,27]. 

The ash content of the avocado-seed flours cv. Hass and landrace (2.40 and 2.52%, respectively) were similar to the values reported by Mahawan, et al. [21] for flour cv. Hass (2.83%). Otherwise, the moisture content in flour from avocado cv. Hass and landrace was 6.68 and 6.94%, respectively. These results are within the range allowed by the Official Mexican Standard NOM-247-SSA1-2008 [34] for commercial flours (<15%). Moreover, the moisture content of both starches was 6.4%, 3.4-fold minor compared with the value allowed in native commercial starches (<20%) [35]. The lower moisture content of avocado starch suggests a low amylopectin content, which is responsible for a higher water retention capacity due to its greater capacity to form more hydrogen bonds with water molecules [12]. 

On the other hand, in the starches of both cultivars, the protein content (0.40–0.45%) and ash (0.05–0.06%) were similar to those reported by Dos Santos, et al. [28] for avocado-seed starch cv. Daisy (ash = 0.38% and protein = 0.07%). These results indicate that the starch isolation from avocado seeds was successful, mainly due to the low content of proteins and fats, meaning that avocado seeds could become a potential source of high purity starches [36].

The color variables in flours and starches were significantly different; these variations can be attributed to the additional purification processes of the starches. Hence, fibers, proteins, pigments and other impurities may have been removed. This behavior is similar to that reported by Rivera, et al. [18] in the flours (L* = 51, a* = 21, b* = 36) and starches (L* = 70, a* = 18, b* = 28) from avocado seeds. The seed starches of both avocado cultivars showed greater whiteness (brightness, L*) than the flours with values of 86–87 (Table 1), but lower than that reported for starches from non-conventional botanical sources, such as the banana (*Musa paradisiaca* L.) (~96) [37], and conventional ones, such as corn (~98) [38]. Color variables have been widely discussed. Most authors agree that reddish-brown coloration is more intense in flours than in starches, and they have attributed it to the presence of various components [18]. In this regard, Builders, et al. [12] attribute this coloration to the iron (1.44 mg/100 g) present in the biopolymeric materials. In contrast, Lubis, et al. [39] attribute this coloration to the high presence of phenolic compounds, such as 3,4 dihydroxyphenylalanine, in the seed that cause an enzymatic browning reaction due to oxidation. Several authors mentioned that avocado-seed starches tend to produce opaque gels due to their characteristic coloration [10,13,28], an attribute that could have potential in the formulation of products that do not require transparency, such as soups, sauces and creams, among others [40].

The apparent amylose content for avocado flours and seed starches in both cultivars is shown in Table 1. The apparent amylose content in flours was 17.46 and 19.73% for cv. Hass and landrace, respectively, while it was significantly higher in the starches with values of 42.25 and 48.02% for cv. Hass and landrace, respectively. Therefore, the landrace cultivar stands out for its high content of apparent amylose in flours and starches. The amylose content greatly influences the physicochemical, thermal and functional properties of starches, especially since they play a fundamental role in the gelatinization and paste formation process [41].

The amylose contents in our starches were similar to those reported by Cornelia and Christianti [14] in the starch from the avocado seeds of cv. Hass (42%) but higher than those recorded for avocado starches of cv. Hass and cv. Daysi (14–32%) [12,13,28]. The Hass cultivar has been widely studied. However, despite being the same cultivar, differences in composition have been reported, mainly in the apparent amylose content 14, 32 and 42% [12,13,14]; thus, it is suggested a high polymorphic diversity in the same cultivar [42]. Agama, et al. [43] these variations to the genetic factors of each cultivar, which are responsible for the amylose and amylopectin biosynthesis. 

### 2.2. Shape and Size Distribution by Scanning Electron Microscopy (SEM)

**Figure 1:**
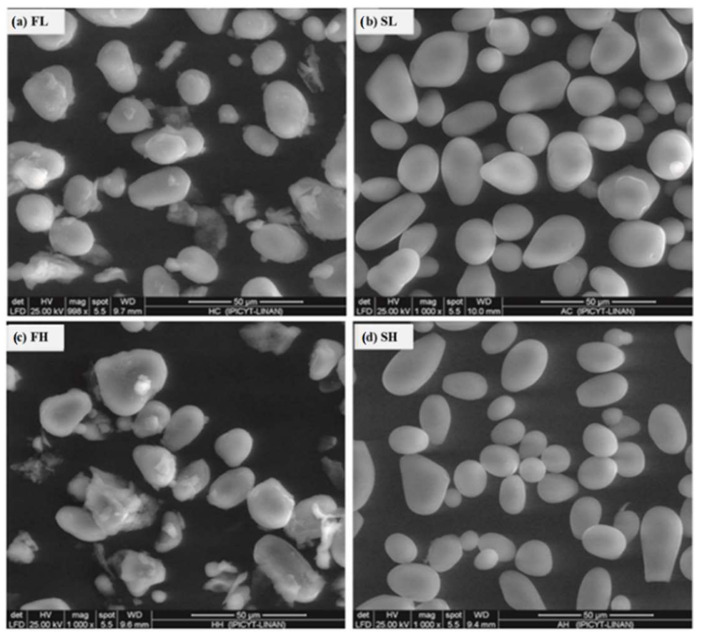
SEM micrographs of flours and starch from avocado seeds. (**a**) FL, landrace flour; (**b**) SL, landrace starch; (**c**) FH Hass flour; (**d**) SH, Hass starch.

The micrographs obtained by SEM analysis in the samples are shown in Figure 1. In the flours, granules with spherical and oval shapes were observed in the Hass and landrace cultivars, respectively (Figure 1a,c). These were found covered by sheets, presumably composed of proteins, lipids and fibers [18,44]. Similar results have been reported by Rivera, et al. [18].

With respect to starch granule size, the results showed significant differences (*p* < 0.05) between cultivars. Avocado flour from avocado seed cv. Hass showed a trimodal distribution (1–20, 21–40 and 46–60 µm) with an average size of 17 µm, while the granules of flour from landrace avocado seed exhibited a monomodal distribution (6–20 µm), with an average size of 19 μm. Similar results to those found in flour cv. Hass has been reported for guineo-banana (*Musa sapientum* L.) flour, with a trimodal distribution (4.17, 16.52 and 42.11 μm) [44]. The particle sizes found in the present study were smaller than those reported by Umaña, et al. [45] for flours from other unconventional botanical sources such as beans (*Phaseolus vulgaris* L.) (37 μm), lentils (*Lens culinaris* Medik.) (30 μm) and banana (99 μm). Espinosa, et al. [46] mentioned that the physicochemical, functional and nutritional properties of the flours are influenced by particle size. In this regard, larger granules show higher viscosity in the formation of pastes, while the smaller ones are easy to digest [44]. 

Compared with the flours, the starches showed similar shapes (oval and spherical), with smooth surfaces without non-starch components such as protein, fiber and lipid (Figure 1b,d) [47]. The granule size distribution of starch cv. Hass was bimodal with a peak at 7–9 µm and another at 15–17 µm with an average size of 18 µm. Various authors reported a similar behavior for avocado-seed starches with sizes ranging from 5 to 30 μm [12,14,18]. On the other hand, the landrace-avocado-seed starch granules showed a monomodal distribution (15–23 μm) with an average size (23.2 μm) within the range reported by other authors for the Hass and Zutano cultivars [10,11,16,24]. Starch granules with oval-to-round forms have been documented by Bet, et al. [11] and Rivera, et al. [18] in avocado-seed starches (cv. Mantequilla and Hass, respectively); however, these starches contained other adhered components, such as fibers and proteins. The differences in sizes and shapes of the starch granules in both cultivars can be attributed to the botanical source, amyloplast morphology and the ripening stage of the fruits [26]. The starch granule sizes significantly influence the functional and physicochemical characteristics of starches. For example, digestibility, water solubility and swelling power tend to decrease with smaller particle sizes [48].

### 2.3. Fourier-Transform Infrared Spectrometry (FTIR)

**Figure 2:**
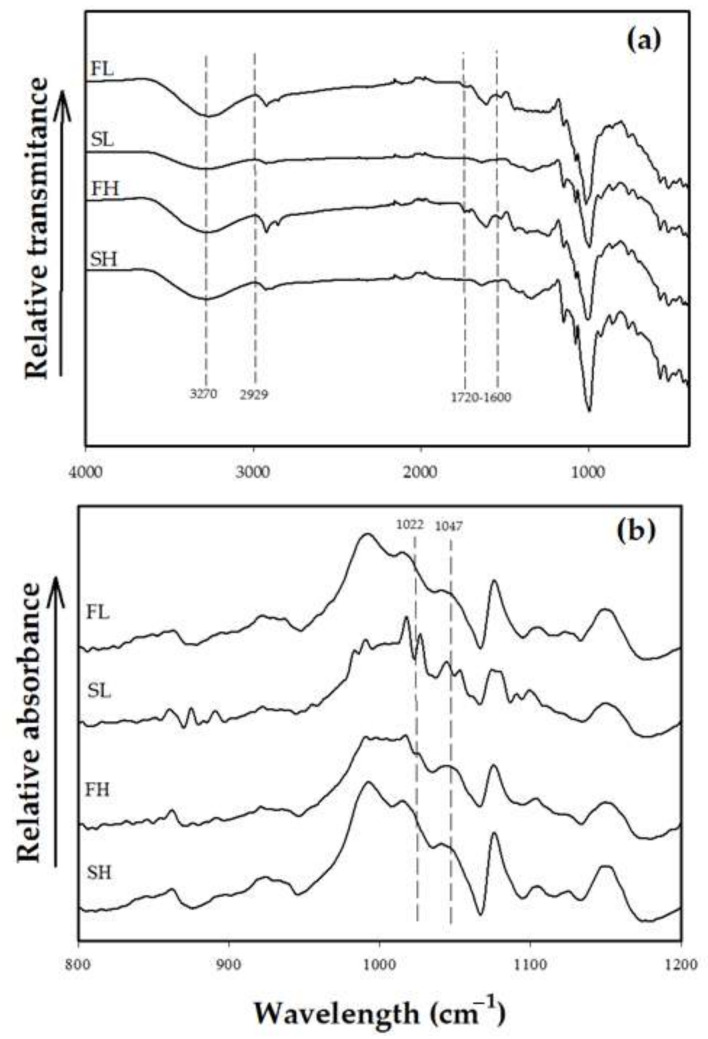
Original (**a**) and deconvoluted (**b**) FTIR spectrum of flours and starches from avocado seeds. FL, landrace flour; SL, landrace starch; FH, Hass flour; SH, Hass starch.

The FTIR spectra of flours and starches are presented in Figure 2a. In all the samples, typical absorption bands of starchy materials were observed. The band stretch around 3400 cm^−1^ is attributed to symmetrical and asymmetric stretches of the O-H bond in the molecules, which has been linked to water molecules [49]. In flours, we observed a band between 1600 and 1720 cm^−1^ that was attributed to the stretching of the C=O bonds, characteristic of the amide I group present in proteins, which is justified by its high protein content (Table 1). The presence of this band has been reported in avocado- and banana-seed flours [18,44]. Further, we observed a peak at 2929 cm^−1^ in the avocado-seed starches of both cultivars, characteristic of the CH_2_ bonds associated with the glucose ring. These results coincide with that reported in avocado-seed starches cv. Hass [50]. 

The spectra of all the samples (Figure 2a) were deconvoluted from 800 to 1200 cm^−1^ and are shown in Figure 2b. Within the fingerprint region (400–1250 cm^−1^), intense characteristic peaks of carbohydrates were recorded; the bands at 1047 and 1022 cm^−1^ correspond to the crystalline (amylopectin) and amorphous (amylose) zones, respectively. The crystallinity ratios obtained from the relationship between the amylose and amylopectin bands indicated that the flour crystallinity index significantly decreased from 0.80 to 0.62 and from 0.82 to 0.63 of flour to starch in cv. Hass and landrace, respectively. This behavior agrees with the results of Rivera, et al. [18], who isolated starch from avocado seed meal and reported that the crystallinity index decreased from 0.68 to 0.60. This behavior was attributed to the partial loss of the molecular interactions between the starch granules and the other flour components. These results suggest that (compared to their starches) flours have a higher degree of molecular order in their structures [51]. In this sense, the molecular reorganization influences some functional properties related to water absorption and viscosity [18]. Further, van Soest, et al. [52] suggest that the high crystallinity index in flours is attributed to the presence of micro cellulose crystals; hence, these results are closely related to the percentage of crystallinity obtained from the X-ray diffraction standards.

### 2.4. X-ray Diffraction (XRD)

**Figure 3:**
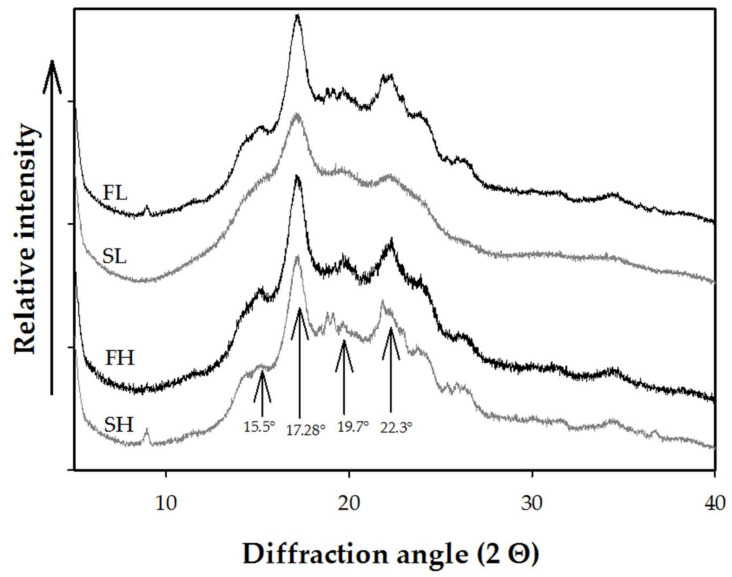
X-ray diffractograms of flours and starches from avocado seeds. FL, landrace flour; SL, landrace starch; FH, Hass flour; SH, Hass starch.

The XRD patterns of flours and starches from avocado seeds are shown in Figure 3. All samples exhibited a B-type crystallinity pattern, with diffraction peaks at angles 15.5°, 17.28°, 19.7° and 22.3° (2θ). Up to date, the information regarding the XRD patterns of avocado-seed flours is null; thus, this would be the first study to characterize them. In this regard, Pelissari, et al. [44], determined the XRD pattern of banana flours and starches without finding significant changes between the peaks within the 2θ angle. In the case of XRD diffractograms of avocado-seed starches cv. Hass and landrace, our results agree with Kahn [16], who describes a pattern of crystallinity of B-type for starch of avocado seed cv. Zutano. However, they differ from Alves, et al. [10] and Lacerda, et al. [53] in avocado-seed starches cv. Hass, which presented an A-type crystallinity pattern with three strong diffraction peaks angles at angles 14.8°, 17.2° and 23.1° (2θ). On the other hand, Bet, et al. [11] reported a C-type crystallinity pattern in avocado starch cv. Mantequilla. These differences in the crystallinity patterns for the same botanical source are dependent on the cultivar, ripening stage and the agro-climatological conditions in which they were cultivated. 

The crystallinity (%) obtained from the diffractograms (Figure 3) is shown in Table 2. The crystallinity varied significantly in all the samples evaluated. Compared with the crystallinity of their flours (23.20 and 25.50% for cv. Hass and cv. landrace, respectively), the starches presented minor crystalline fractions (14.82 and 17.23% for cv. landrace and cv. Hass, respectively). This behavior probably results from the presence of cellulose fibers, which, when presenting ordered lamellae, gave them a crystalline appearance [54]. These results agreed with the crystallinity indexes found, with the ratio of the bands 1022/1047 obtained by FTIR denoting the highest crystallinity of the flours. 

The crystallinity values from avocado-seed flours and starches differ with the behavior reported by Aguirre, et al. [55] for banana flours and starches, in which the lowest crystallinity was observed in the flour. The percentages of crystallinity obtained by XRD of the starches of both cultivars (Table 2) were similar to those reported in avocado-seed starches cv. Hass (9–13%) [24,53] and cv. Mantequilla (14–16%) [11], but lower than cv. Daysi (≥25%) [12]. Compared with the crystallinity (27.91–30.92%) of starches from four banana varieties [56], avocado-seed starches presented lower values. The low crystallinity in our starches could be due to their high amylose content (Table 1) [52].

### 2.5. Differential Scanning Calorimetry (DSC)

The transition temperatures and the gelatinization enthalpy change obtained from thermograms are shown in Table 2. The onset temperature (*T_o_*) of flours varied from 68 to 69 °C, similar to starches (from 66 to 67 °C). These values are higher than those reported for avocado-seed starches cv. Hass and Zutano (62 and 56 °C, respectively) [13,16]. The highest values of heat absorption were recorded at the peak temperature (*T_p_*); it is important to mention that, in this second gelatinization stage, the samples go into a rubbery state, due to the total breakdown of the granules [55].

The *T_p_* between the flours (73.17–73.62 °C) of both cultivars showed no significant differences; however, it was statistically higher than the observed in their starches (70.34–71.14 °C). This behavior is similar to the reported by Rivera, et al. [18], who found that the gelatinization temperatures fluctuated between 67.79 and 76.23 °C and between 65.26 and 74.89 °C in flour and starch of avocado seeds cv. Hass, respectively. These differences can be attributed to the prescence of components such as proteins and lipids in a higher proportion in flours than in starches [44]. The presence of proteins in flours in suspension protects the granules by preventing water entry, while lipids promote the formation of complexes, together with amylose [57].

The *T_p_* values of the starches in both cultivars were lower than those reported for starches of avocado cv. Hass (79.8 °C) [12,14,24] and cv. Mantequilla (76 °C) [10,11]. This behavior is mainly attributed to its high amylose content (Table 1), causing a decrease in the ordered fraction (amylopectin), generating less temperature and energy to perform gelatinization. These results suggest a higher degree of association between the components in the starches of avocado cv. Hass and landrace [58].

Avocado-seed starches cv. Hass and landrace showed significantly higher ∆*H_gel_* values (13.43–11.82 J/g, respectively) than those recorded in their flours (5.92 and 6.55 J/g, respectively). The starches absorbed more energy in the gelatinization process to break the crystalline arrangement of their granules [55]. These results contradict those found by Hoover and Sosulski [58], who mention that, the higher the crystallinity, the greater the energy required for gelatinization to occur, due to a higher order of molecules (see index and percentage of crystallinity in Table 2). This behavior can be due to the partial fusion of the crystals of the ordered region, since the low values of enthalpies in flour may be the consequence of a possible heterogeneity of the ordered structures within the granules [55]. In addition, the experimental conditions during the test, such as the water-sample ratio and the rate (°C/min) of the heating ramp, influence the transition temperatures and enthalpy change [59]. The thermal characterization of materials (especially this polysaccharide) is important because it provides us information to determine the industrial processes to which it can be subjected [60].

### 2.6. Functional Properties

**Figure 4:**
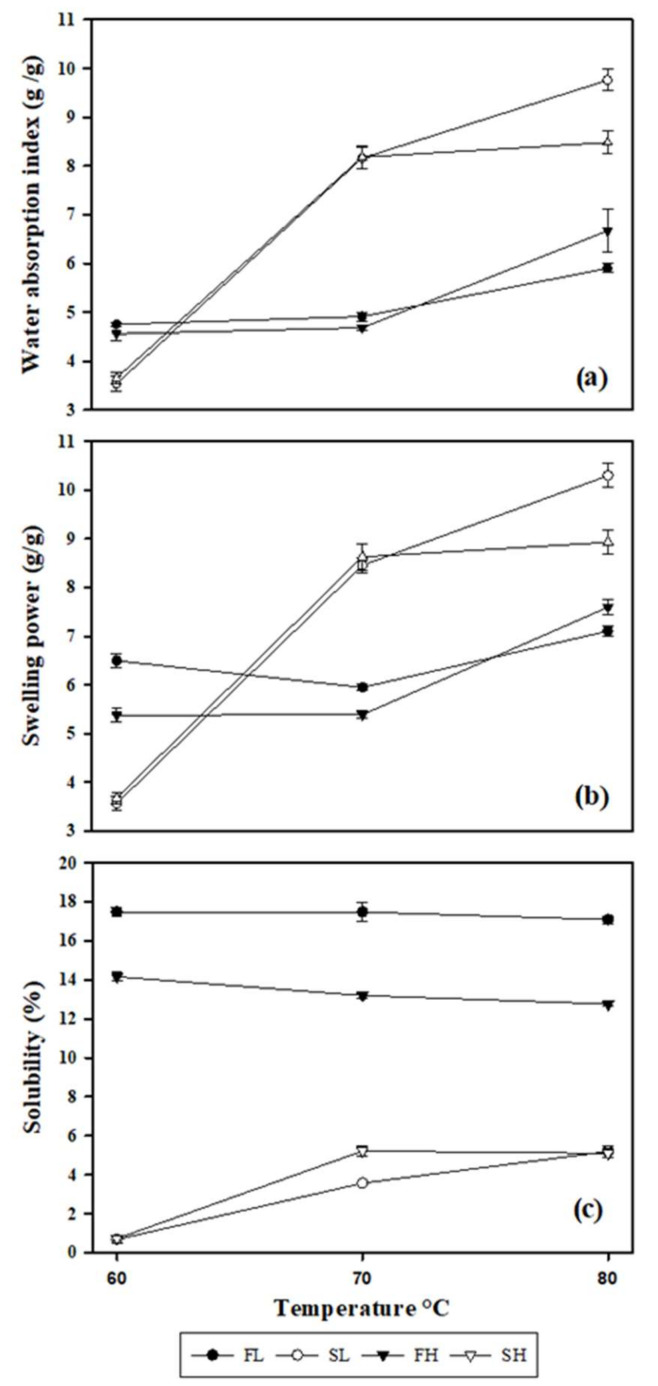
(**a**) Water absorption index (WAI), (**b**) swelling power (SP) and (**c**) water solubility (WS) of flours and starches from avocado seeds. FL, landrace flour; SL, landrace starch; FH, Hass flour; SH, Hass starch.

Figure 4 shows the water absorption index (WAI), water solubility (WS) and swelling power (SP) of the flours and starches from avocado seeds cv. Hass and landrace; these measurements were directly correlated with temperature increases. After the granules are dispersed in water and gradually warmed, they increase their size, due to hydration, until the maximum hydration point is reached (swelling or gelatinization) [61]. 

The WAI and SP are closely related; both properties gradually increased after 70 °C, showing significant differences between the flours and their starches in the Hass and landrace cultivars (Figure 4a,b). On the other hand, at low temperatures (60 °C), for the flours of both cultivars, a higher SP (5.38 and 6.50 g/g) and WAI (4.56 and 4.75 g/g) were observed with respect to their starches (SP = 3.68–3.57 g/g; WAI = 3.66–5.54 g/g). These behaviors can be attributed mainly to non-starchy components (lipids and proteins), especially in flours [51]. These results were similar to those reported by Pelissari, et al. [44] in flour and banana starch cv. Terra (SP, 3.4 g/g; 2.4 g/g, respectively). However, when the temperature increased to 70 and 80 °C, the SP and WAI of the starches were also increased with respect to their flours. Aguirre, et al. [55] mention that, after the granules are dispersed in water and subjected to gradual heating, they tend to increase in size, due to hydration, until reaching the maximum point of hydration (swelling or gelatinization).

According to Chel, et al. [13], the starch granules of avocado seeds cv. Hass tend to absorb water and swell after 70 °C, an effect attributed mainly to the breakdown of intermolecular bridges in amorphous areas. This behavior allows progressive and irreversible absorption of water, promoving a loss of birefringence and an increase in the viscosity of the dispersion.

SP and WAI values (70 and 80 °C) in avocado-seed starches cv. Hass (SP, 8.18–8.48 g/g; WAI, 8.63–8.93 g/g) and landrace (SP, 8.16–9.76 g/g; WAI, 8.16–9.76 g/g) were similar among the cultivars studied [12,13,14]. Nevertheless, the SP values found in the present study were lower than those reported by Paraginski, et al. [62] in corn starch, where a high SP (12.48 g/g) was observed at temperatures above 90 °C. This phenomenon has been attributed to a high amylopectin content in its highly branched structure, allowing a higher water-holding capacity. At the same time, amylose inhibits the swelling of the granules [63]. 

The variation in the functional properties of starches may be due to the degree of coupling of the hydroxyl groups to form hydrogen bonds between the starch chains. In addition, the increase in SP and WAI are associated with increased leaching and solubility of amylose due to the loss of the crystal structure of starches [58]. 

On the another hand, significant differences were observed in the WS of the flours and starches of both avocado cultivars (Figure 4c). The avocado-seed flours cv. Hass (12–14%) and landrace (~17%) showed higher solubility than their starches, with landrace avocado seed being the most soluble at the temperatures evaluated. The high solubility values in flours are mainly attributed to the presence of high protein (5.2%) and lipid (1–2.4%) contents (Table 1) [44]. However, according to Ceballos and Montoya [27], the high solubility of avocado seed meal (cv. Papelillo and Trinidad) was due to their high content of soluble carbohydrates (CHOS, 17–35%). The high WS is desirable in agro-biotechnological processes, such as the microencapsulation of entomopathogenic microorganisms for pest control [64]; therefore, the high solubility of avocado-seed flours makes them potential candidates. 

Avocado-seed starches cv. Hass and landrace showed a low solubility compared to their flours. Nonetheless, in contrast to their flours, when increasing the temperature from 60 to 70 °C, a tendency to slightly increase the WS was observed (from 0.73 to 5.22% and from 0.68 to 3.57%, respectively). At 80 °C, no significant increase in its solubility (5.10–5.20%) was observed, presumably due to the interruption of the granule structure caused by the lixivation of amylose. This behavior has been widely documented in starches from various botanical sources [44]. 

These results were lower than those reported by Chel, et al. [13] for the native starch of avocado seed cv. Hass (WS = 19.7–20.6%); despite their low amylose content (15%), they present the highest percentage of solubility recorded in this botanical source. On the other hand, Cornelia and Christianti [14] observed similar values regarding the apparent amylose content in the starch of avocado seeds cv. Hass (≥42%; Table 1), but its WS is twice as high as the registered in the present study (12.57%). Thus, these variations in WS of starch can be explained by structural differences in the amylose and amylopectin of the granules, such as chain length and molecular-weight distribution [13]. Moreover, the results found in the present research are similar to those recently reported by Alves, et al. [10] and Dos Santos, et al. [28], who found a WS of 0.3–0.45% and 0.2–5.2% in starches from avocado seeds of cv. Mantequilla and Daysi, respectively. These results support the idea that the functional properties, such as WS, WAI and SP, are strongly influenced by the botanical source with differences even between cultivars, mainly depending on their cultivation conditions [10,13,14,28].

### 2.7. Rheological Properties

#### 2.7.1. Flow Curves

In Table 3, the rheological variables of flours and starches from avocado seeds of cv. Hass and landrace are shown. The consistency coefficient (*k*) was significantly low in flours, and this could be due to its lower amylose content and higher presence of components such as proteins and lipids [18], which form insoluble complexes, preventing the penetration of water into granules during swelling and gelatinization processes [65]. Nonetheless, both the flour and the starch of landrace avocado seeds presented higher values in *k* than those obtained in the cv. Hass. This result could be attributed to their higher amylose content (Table 1) [65,66].

**Figure 5:**
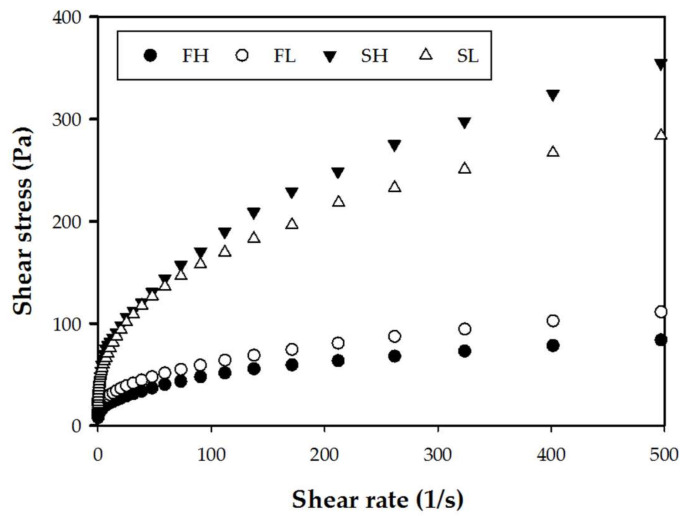
Flow curves for flours and starches isolated from avocado seeds. FL, landrace flour; SL, landrace starch; FH, Hass flour; SH, Hass starch.

The *k* (an analogy of viscosity) of both flours was lower (1.08–1.22 Pa∙s^n^) compared to their starches (1.53–1.55 Pa∙s^n^ cv. Hass and landrace, respectively), which can be related to their low water-absorption capacity (Figure 4a), causing an excess of available water in the solutions [51]. This behavior is similar to that reported by Rivera, et al. [18], who used a Rapid Viscosity Analyzer (RVA) to analyze the viscosity of avocado cv. Hass, finding that the viscosity peak in the flour (2816 Pa∙s) of avocado seeds was lower than the avocado starch (6188 Pa∙s). Compared with the flours, the *k* values of the starches were high, showing that the dispersions are less sensitive to cut (Figure 5). This behavior has been documented in the flours and starches of banana cv. Dominican and Colombian [67,68]. However, the results obtained in avocado-seed starches were lower than those reported by Casarrubias, et al. [69] for starches of barley, corn, banana and mango (Var. *Tommy atkins*) (2.42, 3.42, 6.34 and 14.42 Pa∙s^n^, respectively), showing better qualities for pasta formation.

After adjusting the data to the Ostwal–de Waele model (Power Law) (R^2^ ≥ 0.98), we found that all the dispersions of flour and starches had a flow behavior index (*n*) less than 1, defining them as non-Newtonians fluids with a pseudoplastic type behavior (*n* < 1). Furthermore, the tendency to flow was greater (Figure 5) in flours with lower amylose content (Table 1), since it tends to flow under a melting state [70]. Similar results were reported in barley, corn, mango and banana starches (*n* < 0.23); thus, the botanical source has no relationship with the value of n, since all starches presented similar values [69]. To date, there is no information on the flow behavior of avocado-seed flours, so the results reported in this study would be the first.

#### 2.7.2. Viscoelastic Tests

**Figure 6:**
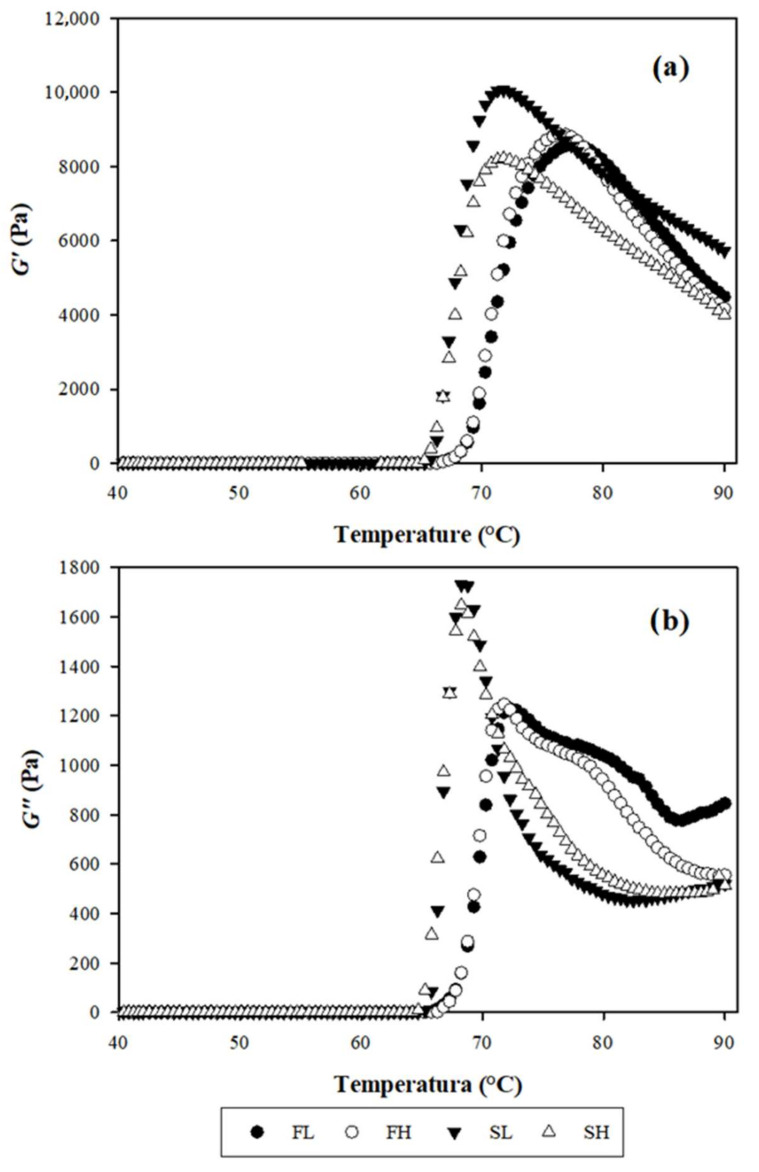
(**a**) Storage modulus (*G′*) and (**b**) loss modulus (*G″*) of flours and starches gels from avocado seeds. FL, landrace flour; SL, landrace starch; FH, Hass flour; SH, Hass starch.

The viscoelastic properties of flours and starches from avocado seeds of cv. Hass and landrace during their heating are shown in Figure 6 and Table 4. The temperature at which *G’* (elastic modulus) was higher during heating (T*G’*) was significantly different between flours from avocado seeds of cv. Hass and landrace (76.53 and 77.82 °C, respectively), followed by their starches (71.63 °C); this agrees with the *Tp* of all the materials (Table 2). These trends have been discussed by Hagenimana, et al. [71] in rice flours and starches (*Oryza sativa* L.) and native corn starch [60]. The T*G’* found in the flours of both cultivars were higher than those registered in corn starches (T*G’* = 67.1–71.2 °C) [60], suggesting that the corn starch is less resistant to heat and to mechanical cutting, so it is more susceptible to loss of viscosity.

According to the values for *G′* and *G″*, the flours and the starches of both cultivars presented a more elastic than viscous behavior (*G′* > *G″*). These results agreed with the reported for flours and starches in various botanical sources, including avocado seeds [13,59,70,71]. The elastic behaviors of our materials give them a great potential to be used as additives in gel-type foods, providing a soft texture, and remaining soft even at low temperatures. These characteristics of avocado-seed starches have potential applications in products such as baby food, sauces, bread products, jellies, sweets and sausages [13]. 

Flours and starches showed no significant differences in module *G′*; however, a decreasing numerical order was observed: SL > FL > FH > SH (Table 4). These differences may be influenced by the granular structure of the materials [71]. Our results were superior to those reported by Hagenimana, et al. [71] in different rice cultivars (*G′* = 240–2947 Pa), but lower than those obtained for corn starches (*G′* = 10,840–14,200 Pa; *G″* = 943–1237 Pa) [60]. 

The values found in the present study for the tangent δ were 0.14 in flours and 0.18 to 0.21 for starches. In this regard, values of tan δ close to zero have been associated with starchy materials, from which weak gels are obtained. Further, the tan δ values in the starches of avocado cv. Hass and landrace were statistically similar (Table 4). The tan δ values in starches were similar to those reported by Chel, et al. [13] in avocado-seed starches cv. Hass (0.10–0.18). These values clearly indicate the amorphous structure of the materials. Therefore, it is suggested that its elasticity could provide a smooth structure and would remain so despite the low temperatures; this attribute can be used in the food industry as a texture additive in the manufacture of puree and dressings [14]. In Figure 6, the *G′* and *G″* reach a maximum value, due to the formation of a network of solvated material from the granules. Finally, when the temperature increases, the *G′* and *G″* values gradually decrease as a result of the breakdown of the network [60].

## 3. Materials and Methods

### 3.1. Materials

The samples from each cultivar were harvested at physiological maturity in the orchard “Los aguacatitos” located in Xalisco, Nayarit, Mexico. All the reagents used were analytical grade and purchased from Sigma-Aldrich (Toluca, Estado de Mexico, Mexico).

**Figure 7:**
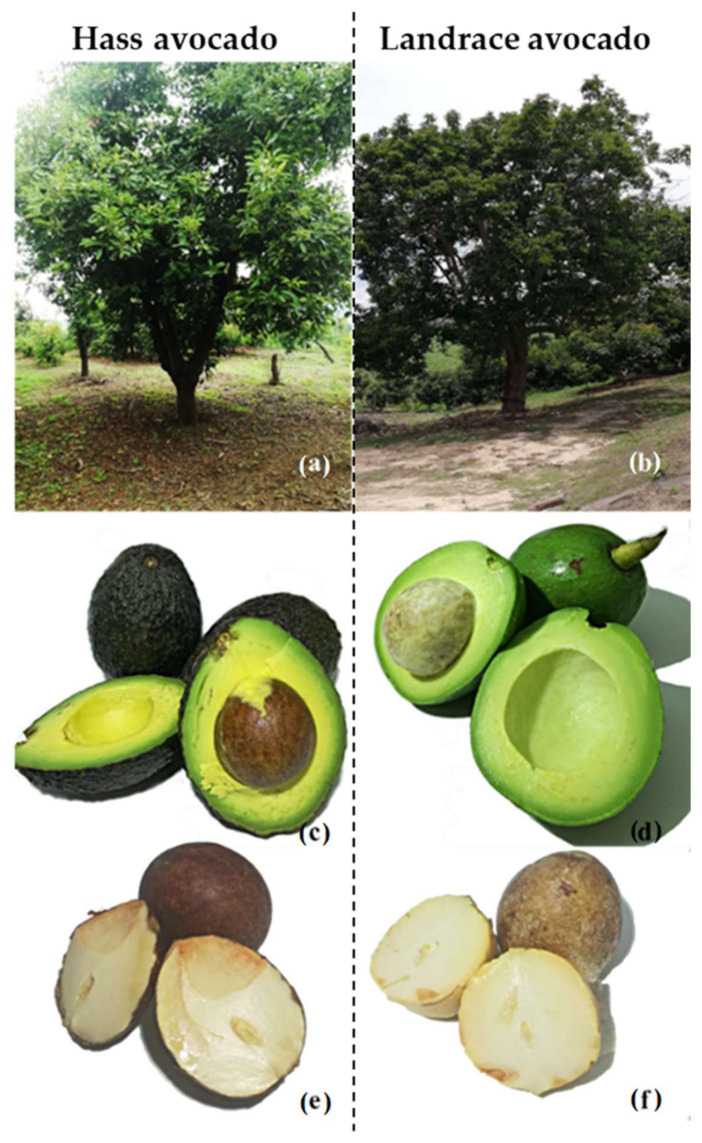
Avocado-tree cultivars (**a**,**b**). Fruit with a longitudinal section showing the internal parts: pulp and seed (**c**,**d**) and seeds (**e**,**f**).

The Hass and landrace avocado cultivars used in this work showed morphological differences in both trees and fruits. Landrace avocado trees (Figure 7b) are taller (~15 m) than cv. Hass (~6 m) (Figure 7a). The average fresh weight of the avocado fruits was 181 g for cv. Hass and 240 g for the landrace, with proportions in the skin, pulp and seed of 66.71, 13.48 and 18.29% for cv. Hass and 64.82, 10.92 and 22.68% for the landrace, respectively.

### 3.2. Obtaining the Flours

The flours were obtained from whole seeds of both avocado cultivars (not oil extraction was realiced). The seeds were washed with a 6% (*v*/*v*) NaClO solution to eliminate pulp residues and thus prevent the proliferation of microorganisms. They were later sliced (~5 mm thick) and immersed in a 1500 ppm sodium metabisulfite (Na_2_S_2_O_5_) solution for 24 h. Seeds were dried in a food dehydrator (Parallex^®^) at 40 °C for 30 h, crushed in an analytical mill (M20, Ika^®^-Werke) and sieved with a 150 µm screen (100 mesh). The flours were stored vacuum-sealed in polyethylene bags and kept refrigerated (4 °C) until further analysis (~20 days).

### 3.3. Starch Isolation

The isolation of starches in both avocado cultivars was carried out according to the methodology of Alves, et al. [10], but with modifications. In this regard, we liquefied 1380 g of flour, with Na_2_S_2_O_5_ added at 1500 ppm in a solid:liquid ratio of 1:5, with an Osterizer commercial blender (Oster^®^, 700 W, capacity 1.5 L), at maximum speed, for 1 min. The suspension was passed through a sieve of 53 µm (270 mesh), and the permeate was allowed to settle for 12 h. Then the supernatant was decanted, and the solids were resuspended in 3 L of distilled water and passed again through a 45 µm screen (325 mesh) to remove the remaining fibers. The obtained dispersion was mixed with 0.05 M NaOH solution and kept under constant stirring (250 rpm) for 60 min and kept at 4 °C for 12 h. The supernatant was decanted, and the solids were resuspended in 1.5 L of distilled water and then centrifuged (14,000× *g*, at 15 °C for 10 min). The supernatant was decanted, and the brown layer that formed on the starch pellet (probably fibers and pigments) was removed. This procedure was performed twice. Subsequently, three washes were carried out with distilled water; the supernatant was decanted, and the sediment was neutralized (pH 7) with 0.05% hydrochloric acid (HCl) and kept at room temperature (24 ± 2 °C) for 2 h, and then the supernatant was discarded. Finally, the sediments were washed with 500 mL of absolute alcohol under constant agitation for 30 min, left to stand for 2 h and then the supernatant was discarded by decantation. The solids were dried in an oven at 40 °C for 24 h and grounded in a mortar until they passed through a sieve of 150 µm (100 mesh). The starch was stored in hermetic polyethylene bags for further analysis. 

### 3.4. Proximate Composition, Tri-Stimulus Color and Amylose Content

The proximate analysis (moisture, fat, ash and crude protein content) of the flours and starches of each cultivar was determined according to the official methods (934.01, 920.39, 942.05 and 954.01, respectively) of the AOAC [72]. The tri-stimulus color was evaluated five times with a Minolta CR-300 colorimeter Konica (Minolta, Osaka, Japan) that was calibrated with a standard white plate. The measurements were recorded on the CIELAB (L*, a* and b*) scale. The apparent amylose content was determined by using an amylose–amylopectin mixture (0–10 mg amylose) for the calibration curve [73]. Absorbances were measured with an Evolution 300 spectrophotometer (UV–VIS) at a wavelength of 625 nm.

### 3.5. Fourier-Transform Infrared Spectrometry (FTIR)

FTIR spectra of all samples were obtained by using an infrared spectrophotometer (Spectrum Two, Perkin Elmer, MA, USA) equipped with an attenuated total reflectance (ATR) accessory [74]. The spectra were acquired from 450 to 4000 cm^−1^ at a resolution of 4 cm^−1^. For each spectrum, an average of 34 scans was taken. After that, spectra were baseline-corrected in the region of 1200–800 cm^−1^; the half-width and deconvolution factor were 26 cm^−1^ and 2.4, respectively. The absorbance intensity of the bands at 1022 and 1047 cm^−1^ was recorded from the baseline. The 1044/1022 cm^−1^ ratio was used to calculate the crystallinity index of the samples [75].

### 3.6. X-ray Diffraction Analysis (XRD)

X-ray diffractograms of the samples were obtained with an X-ray diffractometer (Rigaku, Smartlab model, Tokyo, Japan), with Cu—K α radiation and a high-speed D/teX detector operating at 40 kV and 44 mA. Data were recorded over a dispersion angle between 5° and 40° (2θ).

### 3.7. Particle Size and Scanning Electron Microscopy (SEM)

Particle size analysis was carried out according to the methodology reported by Bustillos, et al. [60], but with modifications. A suspension at 2% (*w/v*) of each sample in distiller water was prepared. An aliquote (10 µL) of the suspension was analyzed in a light microscope (AxioScope.A1, Carl Zeiss, Jena, Germany) equipped with an AxioCam ICc1 camera. Measurements at 1000× were made on 100 granules per sample, using the ZEN lite Software. The morphology of the granules in the samples was analyzed with a scanning electron microscope ESEM FEI QUANTA 200 (FEI Company, Eindhoven, The Netherlands), according to Gunning, et al. [76]. 

### 3.8. Thermal Properties

Thermal analysis of the flour and starches was evaluated by using a differential scanning calorimeter (DSC) model 4000 (Perkin Elmer, MA, USA) according to the method described by Narváez, et al. [77]. Samples of 2 mg dry weight (dw) were weighted in an aluminum pan, and 7 µL of distilled water was added. The pan was hermetically sealed and allowed to stand for 1 h to allow hydration of the granules. After that, the pan and the reference (empty pan) were heated at a rate of 5 °C/min, from 30 to 120 °C. The onset temperature (*T_o_*), peak temperature (*T_p_*), conclusion temperature (*T_c_*) and the enthalpy of gelatinization (∆*H_gel_*) were calculated from the obtained thermograms. 

### 3.9. Functional Properties

The water absorption index (WAI, g/g on a dry weight basis), swelling power (SP, g/g on a dry weight basis) and water solubility (S, %) of flours and starches were evaluated at 60, 70 and 80 °C, according to the method reported by Anderson [78], but with modifications. Samples of 0.444 g (dw) were weighed in 15 mL polypropylene tubes, (Corning ^®^) and 10 mL of preheated distilled water was added to them at the temperatures described above. The samples in suspension were placed in a water bath for 30 min, separately, at a specific temperature (60, 70 and 80 °C). After the start of the heating process, the tubes were shaken in a vortex for 1 min at 10 and 20 min. The suspensions were centrifuged at 6500× *g*, at room temperature (~24 °C), for 30 min. The supernatant with the soluble material was placed in a beaker to dry at 100 °C for 4 h; the tube containing the gelled insoluble material was immediately weighed. The calculations were carried out based on the following equations:

(1) $$\mathrm{WAI}\;(g/g)=\frac{\mathrm{Weight}\;\mathrm{of}\;\mathrm{the}\;\mathrm{gel}\;(g)}{\mathrm{Sample}\;(g)}$$

(2) $$\mathrm{SP}\;\left(g/g\right)=\frac{\mathrm{Weight}\;\mathrm{of}\;\mathrm{the}\;\mathrm{gel}\;(g)}{\mathrm{Samplet}\;\left(g\right)-\mathrm{soluble}\;\mathrm{weight}\;(g)}$$

(3) $$\mathrm{SW}\;(\%)=\frac{\mathrm{Weight}\;\mathrm{of}\;\mathrm{soluble}\;(g)}{\mathrm{Sample}\;\left(g\right)}\;\times \;100\;$$

### 3.10. Rheological Properties

#### 3.10.1. Flow Properties

Flow properties were determined according to the methodology reported by Sánchez, et al. [79], but with modifications. We prepared dispersions of each sample (5% *w*/*w*, dw), and these were placed on the heating Peltier plate in a rheometer (AR 1500ex, TA Instruments, New Castle city, DE, USA) equipped with a parallel system plate (Ø = 60 mm, gap = 1000 µm). The parallel plates were covered with a solvent trap with mineral oil to minimize moisture loss during the test. The samples were cooked in situ between the parallel system plate at constant shear rate and heating-cooling rate of 50 s^−1^ and 2.5 °C/min, respectively. The samples were heated from 25 to 90 °C and held at 90 °C for 10 min. Subsequently, the samples were cooled to 60 °C. At this temperature, three rotational sweeps were carried out, two consecutive sweeps (one ascending and one descending) at a shear rate of 0.06 to 500 s^−1^ and the third ascending from 1 to 500 s^−1^. The data obtained from the final cycle were fitted to the Power Law model proposed by Ostwal–de Waele:

(4) $$\tau =k{\dot{\gamma }}^{n}\;$$

where τ is the share stress (Pa), *k* consistency coefficient (Pa s^n^), $\dot{\gamma }$ shear rate (1/s) and *n* the flow behavior index (dimensionless). All measurements were made in triplicate.

#### 3.10.2. Viscoelastic Properties

The storage (*G′*) and loss modulus (*G″*) were measured within the linear viscoelastic region (LVR), according to Singh, et al. [80], with a rheometer under the configuration previously described. Flour and starch suspensions (20% *w*/*w*, dw) were prepared in distilled water and stirred (Corning PC 420) for 10 min. The suspension was deposited on the Peltier plate surface preheated to 40 °C, covered with a trap and mineral oil. Subsequently, the sample was heated from 40 to 90 °C and maintained at 90 °C for 10 min. After this treatment, the suspension was cooled from 90 to 25 °C. Both processes were performed at a constant heating–cooling rate of 2.5 °C/min. Changes in *G′* and *G″* during heating–cooling were evaluated with a frequency and strain of 1 Hz and 0.5%, respectively.

### 3.11. Statistical Analysis

The results were analyzed under a completely randomized experimental design with a sample size of at least three replicates for each experimental analysis (*n* ≥ 3), and an analysis of variance (ANOVA) was used. Tukey’s test (*p* ≤ 0.05) was used when ANOVA found significant statistical differences. Statistical analysis was performed with Statistical Minitab^®^ 18.1 software (SCIENTEC, State College, PA, USA). 

## 4. Conclusions

Avocado seeds are considered an agro-industrial waste and source of contamination. However, these by-products have great potential for obtaining unconventional flours and starches. The flours of avocado seeds from cultivars Hass and landrace presented high lipid contents and a not negligible protein content. The isolated starches showed a pinkish-brown coloration with oval and spherical granules that were free of impurities. Compared with the flours, the high apparent amylose content in the starches gives them excellent functional properties in WAI and SP. The high amylose values and absence of other impurities evidenced a lower crystallinity in starches than in their flours. The gelatinization temperatures of avocado-seed starches suggest their possible application for the manufacture of puddings or sauces for which high processing temperatures are not required. All the materials obtained from avocado seeds behaved as pseudoplastic materials. In addition, the dynamic oscillatory test revealed that flour and starch pastes were more elastic than viscous. Although the properties of the starches were very similar, the peak gelatinization temperature was higher in the starch from landrace avocado seeds. In general, the starchy materials obtained from avocado seeds have characteristics that give them great potential to be used in the food industry and agro-biotechnological processes.

## Figures and Tables

**Table 1:** Physicochemical properties of flours and starches native from landrace avocado seeds and Hass.

Source	Moisture (%)	Proteins (%)	Lipids (%)	Ash (%)	Yield (%)	Apparent Amylose (%)	L*	a*	b*
FL	6.94 ± 0.03 ^a^	5.29 ± 0.20 ^a^	1.03 ± 0.06 ^b^	2.52 ± 0.01 ^ab^	41.56 ± 1.85 ^a^	19.73 ± 0.50 ^c^	78.41 ± 0.06 ^d^	6.91 ± 0.06 ^a^	17.34 ± 0.09 ^a^
FH	6.68 ± 0.06 ^ab^	5.23 ± 0.09 ^a^	2.42 ± 0.11 ^a^	2.40 ± 0.23 ^a^	46.86 ± 2.09 ^a^	17.46 ± 0.63 ^c^	79.73 ± 0.05 ^c^	6.12 ± 0.15 ^b^	15.58 ± 0.06 ^b^
SL	6.40 ± 0.12 ^b^	0.40 ± 0.20 ^b^	0.13 ± 0.03 ^c^	0.05 ± 0.03 ^b^	39.57 ± 3.21 ^a^	48.02 ± 0.24 ^a^	86.92 ± 0.02 ^b^	4.9 ± 0.01 ^c^	10.34 ± 0.14 ^c^
SH	6.42 ± 0.06 ^b^	0.45 ± 0.02 ^b^	0.23 ± 0.08 ^c^	0.06 ± 0.00 ^ab^	35.47 ± 1.97 ^a^	42.25 ± 1.44 ^b^	87.12 ± 0.05 ^a^	4.67 ± 0.02 ^c^	10.03 ± 0.02 ^cd^

FL = flour cultivar landrace; SL = starch cultivar landrace; FH = flour cultivar Hass; SH = starch cultivar Hass. Values with the same letter within columns, are not significantly different according to the Tukey test (*p* = 0.05).

**Table 2:** Thermal variables of flours and starches native from landrace avocado seeds and Hass.

Source	*T_o_* (°C)	*T_p_* (°C)	*T_e_* (°C)	∆*H_gel_* (J/g)	Crystallinity (%)	Crystallinity Index
FL	68.83 ± 0. 21 ^a^	73.62 ± 0.14 ^a^	79.53 ± 0.89 ^a^	6.55 ± 0.33 ^b^	25.50 ± 0.17 ^a^	0.82 ± 0.01 ^a^
FH	69.26 ± 0.16 ^a^	73.17 ± 0.07 ^a^	78.47 ± 0.35 ^ab^	5.92 ± 0.77 ^b^	23.20 ± 0.06 ^b^	0.80 ± 0.01 ^a^
SL	67.21 ± 0.35 ^b^	71.14 ± 0.35 ^b^	77.48 ± 0.42 ^bc^	11.82 ± 0.89 ^a^	14.82 ± 0.38 ^d^	0.63 ± 0.01 ^b^
SH	66.73 ± 0.29 ^b^	70.34 ± 0.18 ^c^	76.57 ± 0.65 ^c^	13.43 ± 1.14 ^a^	17.23 ± 0.08 ^c^	0.62 ± 0.01 ^b^

FL = flour cultivar landrace; SL = starch cultivar landrace; FH = flour cultivar Hass; SH = starch cultivar Hass. Values with the same letter within columns are not significantly different, according to the Tukey test (*p* = 0.05). *T_o_* = onset temperature; *T_p_* = peak temperature; *T_e_* = end temperature; Δ*H_gel_* = enthalpy of gelatinization.

**Table 3:** Rheological variables adjusted to the model of Power Law ($\tau =k{\dot{\gamma }}^{n}$) for flour and starches from landrace avocado seeds and Hass.

Source	*n*	*k* (Pa∙s*^n^*)	R^2^
FL	0.28 ± 0.00 ^b^	1.22 ± 0.03 ^b^	0.99 ± 0.00
FH	0.29 ± 0.01 ^b^	1.08 ± 0.07 ^b^	0.98 ± 0.00
SL	0.33 ± 0.01 ^ab^	1.55 ± 0.06 ^a^	0.99 ± 0.00
SH	0.36 ± 0.00 ^a^	1.53 ± 0.00 ^a^	0.99 ± 0.00

Arithmetic mean ± standard error. Values with the same literal between columns are statistically equal according to Tukey’s test (*p* = 0.05); *n* = flow behavior index, *k* = consistency coefficient, and R^2^ = coefficient of determination. FL, landrace flour; SL, landrace starch; FH, Hass flour; SH, Hass starch.

**Table 4:** Rheological properties flours and starches from landrace avocado seeds and Hass during heating.

Source	T*G′*	Peak *G′* (Pa)	Peak *G″* (Pa)	Peak tan δ
FL	77.82 ± 0.96 ^a^	8713.33 ± 424.76 ^a^	1254 ± 89.63 ^a^	0.14 ± 0.00 ^b^
FH	76.53 ± 0.47 ^a^	8893 ± 349.50 ^a^	1251 ± 107.66 ^a^	0.14 ± 0.01 ^b^
SL	71.63 ± 0.44 ^b^	10,105.67 ± 454.15 ^a^	1788.00 ± 179.10 ^a^	0.18 ± 0.01 ^ab^
SH	71.63 ± 0.17 ^b^	8225.00 ± 918.16 ^a^	1684.33 ± 148.38 ^a^	0.21 ± 0.02 ^a^

Arithmetic mean ± standard error. Values with the same literal between columns are statistically equal according to Tukey’s test (*p* = 0.05); *n* = flow behavior index, *k* = consistency coefficient, and R^2^ = coefficient of determination. FL, landrace flour; SL, landrace starch; FH, Hass flour; SH, Hass starch.

## Data Availability

Data is contained within the article.

## Sample Availability

Samples of the compounds are not available from the authors.
